# Retraction: Corrigendum: Design, synthesis, and biological evaluation of (E)-N'-((1-chloro-3,4-dihydronaphthalen-2-yl)methylene)benzohydrazide derivatives as anti-prostate cancer agents

**DOI:** 10.3389/fchem.2023.1332618

**Published:** 2023-11-08

**Authors:** Frontiers Editorial Office

The journal and Chief Editors retract the 26 November 2019 article cited above.

Following publication, concerns were raised regarding the integrity of some of the images in the article. After a corrigendum was published, additional concerns were identified, and a further investigation was conducted in line with Frontiers’ policies. The images were determined to have been manipulated, and the authors were unable to provide the raw data for the images in question. As a result, the data and conclusions of the article have been deemed unreliable and the article is therefore retracted.

The retraction of the article was approved by the Chief Editor of Frontiers in Chemistry and the Chief Executive Editor of Frontiers. The authors did not respond to the retraction.

